# Postoperative Opioid Prescription Size and Patient-reported Consumption Following Gender-affirming Mastectomy: A Prospective Survey Study

**DOI:** 10.1097/GOX.0000000000007023

**Published:** 2025-08-04

**Authors:** Kumaran Arivoli, Gabriela J. Kim, Brooke Kenney, Caleb Haley, Megan Lane, Christopher Breuler, Shane D. Morrison, Jennifer Waljee, Jessica J. Hsu

**Affiliations:** From the * University of Michigan Medical School, Ann Arbor, MI; † Michigan Opioid Prescribing Engagement Network, University of Michigan, Ann Arbor, MI; ‡ Section of Plastic Surgery, Department of Surgery, University of Michigan, Ann Arbor, MI; § Division of Plastic Surgery, Department of Surgery, University of Washington, Seattle, WA; ¶ Department of Urology, University of Washington, Seattle, WA.

## Abstract

**Background:**

Aligning opioid prescribing with patient needs is important for managing pain while minimizing risks. However, little is known about opioid prescribing after gender-affirming procedures. Given that gender-diverse patients are at higher risk for poor opioid-related outcomes, creating guidelines centered on patient-reported opioid use and pain experiences is critical. We conducted a prospective survey study examining opioid prescribing and consumption after gender-affirming mastectomy (GAM).

**Methods:**

Patients who underwent GAM from February to September 2021 were identified. Electronic medical record data were collected, including opioid prescription and refill amounts. Patient-reported outcomes including opioid use, nonopioid adjunct use, pain rating 1 week postoperatively, and pain management satisfaction were collected via telephone surveys 2–4 weeks postoperatively. Descriptive statistics and bivariate analyses compared outcomes between opioid consumers and nonconsumers.

**Results:**

Of 115 patients, 72 responded with complete data (62.6% response rate). Sixty-three individuals (87.5%) consumed opioids and 9 (12.5%) did not consume opioids. The median (interquartile range) opioid prescription quantity was 15 (12–17) tablets. The median (interquartile range) consumption was 10 (3–14.5) tablets (*P* < 0.001). Most patients (63.4%) felt their prescription amount was appropriate, whereas 28.2% said it was too much. In all, 94.4% used nonopioid adjuncts, and 100% were satisfied with their postoperative pain management.

**Conclusions:**

Most patients undergoing GAM reported using opioids, with an average of 10 pills, and were satisfied with their postoperative pain control. Going forward, surgeons should consider tailoring prescribing to patient needs and providing nonopioid alternatives when possible.

**Takeaways**

**Question:** What are common opioid prescription and consumption patterns following gender-affirming mastectomy (GAM), and how can these data guide effective pain management while minimizing risk?

**Findings:** Although opioid consumption following GAM was common (87.5%), most patients consumed fewer opioids than prescribed, with a median consumption of 10 tablets (5 mg oxycodone) and a median prescription size of 15 tablets. In all, 94.4% used nonopioid adjuncts, and all were satisfied with postoperative pain management.

**Meaning:** Surgeons can safely reduce opioid prescriptions following GAM without compromising patient satisfaction by using a patient-centered approach, including preoperative discussions regarding pain expectations and nonopioid analgesic strategies.

## INTRODUCTION

Opioid analgesics are important components of postoperative pain management. However, opioid prescribing during surgical care often occurs in the absence of guidelines and frequently in excess. This excess prescribing has detrimental consequences and poses a greater risk of persistent postoperative opioid use, with approximately 6% of opioid-naive patients continuing to use opioids for more than 3 months after both major and minor procedures.^1^ Additionally, evidence suggests that postoperative opioid consumption increases with higher opioid prescription quantities.^2^ Moreover, excessive prescribing may allow for opioids to be diverted to unintended users, heightening the risk of misuse and abuse. Given these risks, developing guidelines to align postoperative prescribing with patient needs is critical to prevent unintended harm.

Although previous studies have examined opioid prescription patterns and use across a wide range of surgical procedures, data remain limited regarding gender-affirming procedures. Additionally, there is evidence to suggest that gender-diverse patients are at higher risk of substance abuse and prescription pain medication misuse compared with cisgender patients, as a strategy to cope with discrimination.^3–6^ Given that rates of gender-affirming procedures have nearly tripled from 2016 to 2019,^7^ optimization of postoperative opioid use for this patient population is essential. There are currently no widely accepted opioid prescribing guidelines for gender-affirming mastectomy (GAM), which emphasizes the need to better understand opioid use among gender-diverse patients after this procedure.

In that context, a prospective survey study was performed to examine postoperative opioid prescription patterns and consumption following elective GAM, the most common gender-affirming procedure.^7–10^ This was done at a tertiary care center that serves as the longest continually operating gender-affirming surgical unit at a US academic medical center.^11^ Through analysis of patient-reported outcomes from telephone surveys and electronic medical record (EMR) review, our aim was to highlight opioid prescription patterns and consumption.

## METHODS

The Comprehensive Gender Services Program (CGSP) at Michigan Medicine helps gender-diverse individuals access and obtain high-quality gender-affirming medical care. Surgical teams within the CGSP include specialists in plastic surgery, urology, otolaryngology, and gynecology. Patients of the CGSP who met the inclusion criteria were identified through Michigan Medicine’s EMR. This study was deemed exempt by the University of Michigan’s institutional review board as part of a multispecialty quality improvement project to reduce opioid prescribing, and informed consent was not required. Study reporting conforms to the Strengthening the Reporting of Observational Studies in Epidemiology guidelines.^12^

### Study Cohort

Patients 18 years of age or older were surveyed if they underwent GAM at the institution, in either the inpatient or outpatient setting, between February 2021 and September 2021. All patients met World Professional Association for Transgender Health Standards of Care surgical requirements before undergoing GAM.^13^ Surveys were conducted by telephone between 2 and 4 weeks postoperatively, a commonly used timeline for postoperative surveys.^14,15^ A trained member of the project staff provided a verbal explanation of the project, including the purpose, time commitment, inclusion/exclusion criteria, risks and benefits, and confidentiality. Survey topics included postdischarge opioid and adjunct pain medication consumption, opinions regarding opioid prescription amounts, subjective pain score 1 week postoperatively, and overall satisfaction with pain management. Additional exclusions were made for patients who declined to participate, had incomplete survey responses, did not speak English, or had a hospital stay of more than 30 days.

### Study Outcomes and EMR Data Collection

This study had 2 primary outcomes: initial postoperative opioid prescription quantities and patient-reported postoperative opioid consumption. These quantities were converted into oral morphine equivalents (OMEs) and standardized to 5 mg–equivalent oxycodone tablets. When collecting data on initial opioid prescription consumption, allowable responses for opioid consumption were capped at the total quantity prescribed at discharge. For example, if a patient reported consuming from both their initial prescription and a refill prescription, only the consumption amount from the initial prescription was recorded. Our secondary outcome was self-reported pain addressed by the survey item, “average pain rating in the first week after surgery.” This included 5 possible responses of no, minimal, moderate, severe, and worst possible pain. Pain scores between patients who used an opioid and those who did not use an opioid were compared. Pertinent data were extracted from the patient EMR (ie, date of surgery, age, race, gender identity, opioid prescription quantities at discharge, and opioid medication refill status). Further review of information included patient comorbidities (diabetes, obesity, hypertension, previous tobacco use, and current mental health conditions), American Society of Anesthesiologists classification, history of opioid use, use of intraoperative ketorolac, and prescription usage for a mental health condition.

### Statistical Analysis

Patients were included for analysis if they consented to participate in the study, were prescribed opioids postoperatively, and had complete survey response data. Descriptive statistics were calculated for all patients who met the inclusion criteria, and postoperative behavior and sentiments about pain management were also described. Bivariate differences between patients who consumed an opioid postoperatively and those who did not consume an opioid were assessed using the *t* test, Mann-Whitney *U* test, chi-square test, or Fisher exact test when small sample sizes were present. The distribution of OMEs prescribed compared with OMEs consumed was evaluated using the Wilcoxon signed-rank test. All analyses were conducted using SAS version 9.4 (SAS Institute, Inc., Cary, NC). *P* values less than 0.05 were considered statistically significant. Given that the primary aim of this study was to better understand opioid prescription patterns and consumption behavior, a formal power analysis was not conducted.

## RESULTS

### Patient Cohort

**Fig. 1.**
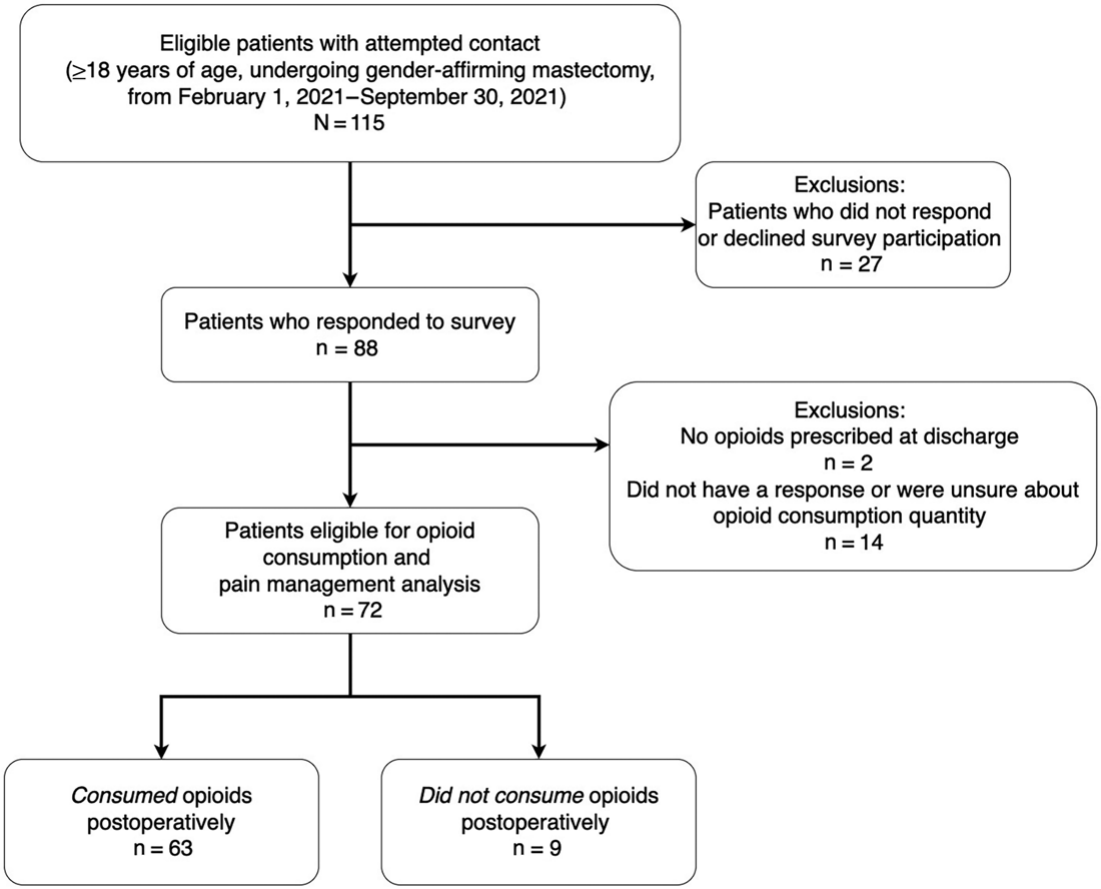
Patient flow diagram for GAM analytical cohort.

Of 115 patients meeting inclusion criteria, 88 agreed to participate in the survey, whereas the remaining 27 patients declined or could not be reached. Of these 88 patients, 14 had incomplete responses to the opioid consumption question, and 2 were not prescribed any opioid medications by patient request. This left 72 patients eligible for the opioid consumption analysis, for a final response rate of 62.6% (Fig. 1). Table 1 displays the study participants’ demographic information. The median (interquartile range [IQR]) age of the cohort was 26 (21–32) years, with 73.6% being White. In all, 70.8% of patients were identified as transgender male, and 25.0% identified as nonbinary. One patient was reported to have consumed opioids in the 30 days, leading up to surgery. Within this cohort, 63 (87.5%) patients consumed opioids postoperatively, and 9 (12.5%) patients did not consume any opioids postoperatively.

**Table 1.** Characteristics of GAM Respondents With a Discharge Opioid Prescription

	No Opioid Consumption (n = 9, 12.5%)	Consumed Opioids (n = 63, 87.5%)	Total (n = 72, 100%)	*P*
Age, median (IQR), y	26 (23–32)	26 (21–32)	26 (21–32)	0.845
Gender identity, n (%)				
Transgender male	7 (77.8)	44 (69.8)	51 (70.8)	0.303
Male	1 (11.1)	2 (3.2)	3 (4.2)	
Nonbinary	1 (11.1)	17 (27)	18 (25)	
Race, n (%)				
White	6 (66.7)	47 (74.6)	53 (73.6)	0.576
Black	2 (22.2)	11 (17.5)	13 (18.1)	
Native Hawaiian/Pacific Islander	0 (0)	0 (0)	0 (0)	
Asian	0 (0)	1 (1.6)	1 (1.4)	
American Indian/Alaskan Native	0 (0)	2 (3.2)	2 (2.8)	
Other	1 (11.1)	2 (3.2)	3 (4.2)	
Inpatient, n (%)	1 (11.1)	5 (7.9)	6 (8.3)	0.565
ASA classification, n (%)				
1	1 (11.1)	12 (19.1)	13 (18.1)	0.258
2	6 (66.7)	47 (74.6)	53 (73.6)	
3	2 (22.2)	4 (6.4)	6 (8.3)	
Comorbidities, n (%)				
Diabetes	0 (0)	5 (7.9)	5 (6.9)	1.00
Obesity	4 (44.4)	32 (50.8)	36 (50)	0.722
Hypertension	2 (22.2)	6 (9.5)	8 (11.1)	0.260
Opioid use 30 d before surgery, n (%)	0 (0)	1 (1.6)	1 (1.4)	1.00
Former tobacco use, n (%)	4 (44.4)	29 (46)	33 (45.8)	0.277
Mental health condition, n (%)	4 (44.4)	48 (76.2)	52 (72.2)	0.104
Prescription use for a mental health condition, n (%)	2 (22.2)	36 (57.1)	38 (52.8)	0.075
Testosterone, n (%)	7 (77.8)	45 (71.4)	52 (72.2)	1.00
Intraoperative Toradol, n (%)	0 (0)	3 (4.8)	3 (4.2)	1.00

ASA, American Society of Anesthesiologists.

### Trends in Opioid Prescribing and Opioid and Nonopioid Analgesic Use

**Fig. 2.**
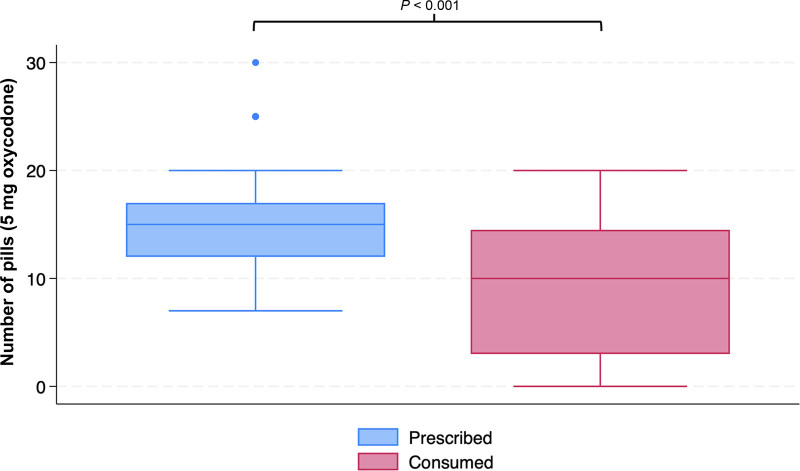
Opioid prescription vs consumption after GAM. The median number of tablets prescribed, standardized to 5 mg oxycodone, was 15 with an IQR of 12–17 tablets. The number of pills consumed was significantly less, with a median of 10 and an IQR of 3–14.5 (*P* < 0.001).

The median (IQR) for the postoperative opioid prescription quantity was 15 (12–17) tablets. The median (IQR) for patient-reported opioid consumption was 10 (3–14.5) tablets (*P* < 0.001) (Fig. 2). In all, 63.8% of patients reported consuming fewer opioids than prescribed. Seventeen (23.6%) patients had their opioid prescription refilled. A total of 70.8% of patients consumed oral acetaminophen as the only other postoperative pain adjunct, whereas 19.4% consumed both oral acetaminophen and ibuprofen as adjuncts. There were no significant differences in the amount of nonopioid pain adjuncts consumed between patients who did and did not consume opioids after surgery (*P* = 0.521) (Table 2).

**Table 2.** Postdischarge Pain Management Experiences of Gender-affirming Surgery Respondents With a Discharge Opioid Prescription

	No Opioid Consumption (n = 9, 12.5%)	Consumed Opioids (n = 63, 87.5%)	Total (n = 72, 100%)	*P*
Refills, count,* n (%)				
0	8 (88.9)	47 (74.6)	47 (76.4)	
1	1 (11.1)	13 (20.6)	14 (19.4)	0.783
2	0 (0)	3 (4.8)	3 (4.2)	
Feelings about quantity of opioid prescribed†, n (%)				
The right amount	3 (37.5)	42 (66.7)	45 (63.4)	
Too much	4 (50)	16 (25.4)	20 (28.2)	0.045
Too little	0 (0)	5 (7.9)	5 (7)	
Unsure	1 (12.5)	0 (0)	1 (1.4)	
Other types of pain adjunct used, n (%)				
Acetaminophen	9 (100)	42 (66.7)	51 (70.8)	
Ibuprofen	0 (0)	3 (4.8)	3 (4.2)	0.521
Both	0 (0)	14 (22.2)	14 (19.4)	
None	0 (0)	4 (6.4)	4 (5.6)	
Average pain rating in the first week after surgery, n (%)				
No pain	3 (33.3)	1 (1.6)	4 (5.6)	
Minimal	4 (44.4)	25 (39.7)	29 (40.3)	
Moderate	2 (22.2)	24 (38.1)	26 (36.1)	0.020
Severe	0 (0)	10 (15.9)	10 (13.9)	
Worst possible pain	0 (0)	3 (4.8)	3 (4.2)	
Pain management since surgery, n (%)				
Worse than expected	8 (88.9)	35 (55.6)	43 (59.7)	
About what was expected	1 (11.1)	24 (38.1)	25 (34.7)	0.238
Better than expected	0 (0)	4 (6.4)	4 (5.6)	
Satisfaction with pain management, n (%)				
1 (not satisfied at all)	0 (0)	0 (0)	0 (0)	
2	0 (0)	0 (0)	0 (0)	
3	0 (0)	0 (0)	0 (0)	0.340
4	0 (0)	12 (19.1)	12 (16.7)	
5 (very satisfied)	9 (100)	51 (81)	60 (83.3)	

* According to the EMR chart review, a refill was submitted for 1 patient who reported to have consumed no opioids.

† One patient did not respond to this question.

### Patient-reported Pain Experiences

Among the total cohort, 63.4% of patients felt that the number of opioids prescribed to them was “the right amount,” 28.2% felt that the prescription amount was “too much,” and 6.9% felt that the prescription amount was “too little.” One week after surgery, 33.3% of patients who did not consume postoperative opioids reported “no pain,” whereas 1.6% of patients who consumed postoperative opioids reported “no pain” (*P* = 0.020). In all, 100% of patients rated their satisfaction with overall postoperative pain management as at least 4 out of 5 (“satisfied”), with the large majority (83.3%) rating it as 5 out of 5 (“very satisfied”). There was no significant difference in satisfaction between patients who consumed opioids and those who did not consume opioids (*P* = 0.340) (Table 2).

## DISCUSSION

In this prospective survey study of patients undergoing GAM, our results demonstrate that postoperatively, most patients use opioid pain medications. Because of this, it is critical to create evidence-based guidelines that align opioid prescribing with patient needs following this common procedure. This is particularly important given that there are currently no opioid prescribing guidelines following GAM. Specifically, most patients consume about 10 pills of 5 mg of oxycodone with a range of 3–15 pills, suggesting that guidelines to direct postoperative prescribing should fall within this range.

Our results also show that there was overwhelming patient satisfaction with our current practice of postoperative pain management. In fact, every patient surveyed rated their overall satisfaction with pain management as at least 4 out of 5, with the majority rating it as 5 out of 5. Additionally, very few patients felt that they did not receive enough opioids postoperatively. Although these findings are encouraging, there may be opportunities to continue to reduce opioid prescribing with the use of both perioperative and postoperative nonopioid adjuncts. Moreover, multiple studies suggest that there is no association between opioid prescription dosing and patient satisfaction.^16–18^ For example, Fry et al^16^ showed that the difference in postoperative pain satisfaction between those receiving 25 OMEs and 750 OMEs was about 2% for patients undergoing common general surgery procedures. Louie et al^17^ found that when prescribing amounts decreased by more than 50%, patient satisfaction did not significantly change. In our study, there was no significant difference in satisfaction between patients who did not consume any postoperative opioids and those who did consume postoperative opioids. However, satisfaction with pain management after surgery is multifaceted and may also reflect other factors, such as the occurrence of complications; patient–provider interactions; and patient expectations, preferences, and previous experiences.

Additionally, we show that most patients consume fewer opioids than prescribed. This discrepancy between opioid prescription patterns and consumption is reflected in various studies outside of gender-affirming surgery.^19–22^ We also found that more than a quarter of patients undergoing GAM felt that the amount of opioid they were prescribed was too high. These findings indicate that surgeons may be overprescribing opioids. However, it is also important to note that some patients may find some comfort in having a postoperative opioid prescription amount that is larger than what they will ultimately need. Pius et al^23^ found that 42% of patients surveyed after surgery expressed the sentiment that they had more opioids than they needed, but not more than they wanted. Despite this, prescribing an excess of opioids results in unused tablets, which can unfortunately lead to diversion into the larger community. Although our study did not specifically assess opioid disposal among surveyed patients, prior work has demonstrated that less than 10% of patients properly dispose of unused opioid medications after surgery.^24^ Thus, calibrating opioid prescribing to actual patient needs is important to minimize the risk of misuse and abuse. Although pain and opioid consumption are normal postoperative experiences after GAM, we hope that our findings can provide evidence to support guidelines that better align opioid prescribing patterns and consumption to minimize these risks. Going forward, improving perioperative education to set pain expectations for patients as well as ensuring an efficient process for patients to request additional opioids if necessary is important to ensure a safe and comfortable recovery.

Prior work assessing postoperative pain outcomes for other surgical procedures has shown that nonopioid analgesia adjuncts serve as an effective method for managing pain with relatively high patient satisfaction.^18,25–27^ Within our cohort, 94.4% of patients used either acetaminophen or ibuprofen as nonopioid pain medication adjuncts. Furthermore, there was no significant difference in the rates of acetaminophen or ibuprofen use when comparing patients who did or did not consume postoperative opioids. This multimodal pain management method may have contributed to the high patient satisfaction observed in our study and underscores the need for further education and patient counseling on possible nonopioid medication options for postoperative recovery.

To date, there is only 1 other study comparing postoperative opioid prescription and consumption patterns for GAM. This was a retrospective study conducted by Robinson et al^14^ that compared opioid outcomes of 3 different breast procedures: oncologic mastectomy, reduction mammoplasty, and GAM. Their results similarly demonstrate a discrepancy in which the number of opioids prescribed was significantly higher than the number of opioids consumed. Specifically, they show that for GAM, the median postoperative consumption amount of 5 mg oxycodone tablets was 15. Given that their findings are similar to this study’s results, prescribing approximately 10–15 pills may be an appropriate amount following GAM. Although the data from our study support this range as a potential prescription guideline that aligns with actual patient use, future research should assess whether prescribing fewer than 10 tablets also achieves adequate pain control and high satisfaction. Our study contributed to this evolving conversation by incorporating patient-reported outcomes that address pain expectations, satisfaction, and prescription quantity perceptions. However, further reductions in postoperative opioid consumption could potentially be accomplished by using strategies such as the use of nonopioid adjuncts, multimodal pain management, patient education, and expectation setting.^28^

There are limitations to this study. First, given the size of our study cohort, we were underpowered to examine the association between risk factors for opioid use, such as comorbid conditions, mental health diagnoses, or surgical complications, and opioid consumption behavior. Further research is needed to determine which characteristics are associated with increased opioid consumption following GAM. This would allow for more individualized, patient-centered prescribing practices. Additionally, this single-institution study may limit generalizability to other institutions with different prescribing patterns and patient populations. Our study also partly relied on survey data, which are subject to recall bias when patients are asked to remember objective quantities of opioid medications they consumed and subjective feelings regarding postoperative pain. We attempted to limit this bias by using a prospective survey design with a relatively short 2- to 4-week follow-up. We additionally excluded patients with incomplete survey responses who were potentially unable to accurately recall postoperative opioid consumption. Of note, we found that 2 patients who did not receive any refills reported consuming more opioids than they were prescribed. This discrepancy may be related to recall bias, or it could be due to patients obtaining extra opioid pills that they were not prescribed. Social desirability bias, where patients tend to underreport socially undesirable behaviors, is another phenomenon that may have affected our results. For example, 1 patient reported consuming 0 OMEs of postoperative opioids; however, based on chart review, they received a refill of their opioid prescription. Social desirability bias, recall bias, or diversion into the community may have contributed to this instance. We attempted to control for social desirability bias by emphasizing patient confidentiality during telephone interviews. Additionally, those conducting the interviews were project team members not involved in the patients’ clinical care.

It is also important to account for confounding pain management modalities. For example, 1 patient in our cohort consumed opioids 30 days before surgery, and 3 patients received intraoperative ketorolac. Additionally, although it is generally the standard practice for the surgeons in our study to use local anesthesia perioperatively, the exact administration method (eg, tumescent, intercostal blocks) varies by surgeon. Within the literature, there are various studies evaluating the effect of different regional blocks on opioid consumption after GAM.^29–31^ However, none have examined the impact of intraoperative ketorolac or opioid-naive versus opioid-tolerant status on postoperative opioid consumption in this patient population, all of which would be interesting areas of exploration for future research. Furthermore, given the heterogeneity of practice patterns, the use of nonopioid pain adjuncts such as acetaminophen and ibuprofen also varied in our sample. Because there was no standardized postoperative pain management protocol at our institution for GAM, nonopioid adjuncts were sometimes prescribed by surgeons and sometimes obtained over the counter by patients. Additionally, postoperative instructions regarding which nonopioid adjuncts patients should use varied by surgeon. All of these factors may have contributed to variation in postoperative opioid consumption and patient satisfaction. Unfortunately, given the variability in obtaining and using nonopioid adjuncts, we were unable to accurately measure or account for this in our analysis.

Finally, during the past 20 years, there has been increased interest in Enhanced Recovery After Surgery (ERAS) protocols as a tool to improve the quality of surgical recovery.^32^ However, ERAS protocols following GAM are currently limited, and there are no ERAS Society guidelines for gender-affirming surgery as a whole.^33^ As such, an ERAS protocol was not used in this study. However, the variation in perioperative pain management seen in our study, including the nonstandard use of nonopioid pain adjuncts and local anesthesia, highlights the opportunity to develop an ERAS protocol to optimize recovery following GAM. Looking toward the future, the results from our study can be used for the development and implementation of these evidence-based protocols. By incorporating patient education, expectation setting, regional anesthesia, and nonopioid adjuncts (eg, acetaminophen, ibuprofen, and gabapentin) into ERAS protocols for GAM, future opioid consumption may be reduced without compromising pain control or satisfaction.

## CONCLUSIONS

To our knowledge, our study represents one of the largest cohorts examining postoperative opioid prescription and consumption in patients undergoing GAM. Our study highlighted 3 findings. First, most patients consume opioid medications after GAM, with 10 tablets of 5 mg of oxycodone being the median amount consumed. Second, there is overwhelming patient satisfaction with postoperative pain management. Finally, most patients consume fewer opioids than they are prescribed. Current trends in opioid outcomes research increasingly focus on the balance between overprescribing, which may lead to misuse or diversion, and underprescribing, which may result in inadequate pain control. Striking this balance is challenging, as individuals vary regarding pain tolerance, personal experiences with different pain management modalities, and preexisting beliefs about opioid medications. Even within our cohort, there was considerable variation in opioid consumption. Therefore, it is important for providers to address pain management in a patient-centered manner through shared decision-making. This includes having preoperative conversations regarding patients’ opinions on opioids, discussing expectations for postoperative pain, optimizing the use of nonopioid pain adjuncts, and ensuring efficient ways for patients to request refills if needed. Using strategies such as these, surgeons performing GAM can decrease their opioid prescription amounts without compromising patient care and satisfaction.

## DISCLOSURES

Dr. Waljee was supported by the Michigan Department of Health and Human Services (E20180672–00). Dr. Haley was supported by the National Institutes of Health (T32 HS000053). The other authors have no financial interest to declare in relation to the content of this article.

## ACKNOWLEDGMENT

Support for this publication was provided by the University of Michigan’s Overdose Prevention Engagement Network and the National Clinician Scholars Program, housed within the Institute for Healthcare Policy and Innovation at the University of Michigan.
